# A Risk Stratification for Patients with Cervical Cancer in Stage IIIC1 of the 2018 FIGO Staging System

**DOI:** 10.1038/s41598-019-57202-3

**Published:** 2020-01-15

**Authors:** Xiaoliang Liu, Weiping Wang, Ke Hu, Fuquan Zhang, Xiaorong Hou, Junfang Yan, Qingyu Meng, Ziqi Zhou, Zheng Miao, Hui Guan, Jiabin Ma, Jing Shen, Hongnan Zhen, Wenhui Wang

**Affiliations:** Department of Radiation Oncology, Peking Union Medical College Hospital, Chinese Academy of Medical Sciences & Peking Union Medical College, Beijing, People’s Republic of China

**Keywords:** Cervical cancer, Cervical cancer

## Abstract

This retrospective study was designed to investigate the heterogeneity of patients with cervical cancer in stage IIIC1 (the 2018 International Federation of Gynecology and Obstetrics staging system, FIGO) and conduct a risk stratification for this group of patients. We reviewed clinical records of 325 patients with stage IIIC1 treated with definitive concurrent chemoradiotherapy in our institute between January 2008 and December 2014. The median follow-up duration was 28.4 months (range: 1.9–114.2 months). The 3-year DFS for the 325 eligible patients was 66.3%. Tumor size of ≥4 cm and number of pelvic lymph node metastasis ≥2 were identified as adverse prognostic factors for disease free survival (DFS) in cervical cancer patients with stage IIIC1 (2018). A risk stratification based on the number of identified prognostic factors for DFS was performed. The 3-year DFS for patients in low-risk (without prognostic factor), intermediate-risk (with one prognostic factor) and high-risk group (with two prognostic factors) was 92.1%, 70.0%, and 51.1%, respectively (P < 0.001). Our study confirms the heterogeneity of patients with cervical cancer in FIGO stage IIIC1 (the 2018 FIGO staging system). Tumor size and number of pelvic lymph node metastasis (PLNM) are significant prognostic factors for DFS in patients with FIGO stage IIIC1. The next revision of FIGO staging system for cervical cancer, especially for stage IIIC1, should focus on tumor size and number of pelvic lymph node metastasis.

## Introduction

Globally, Cervical cancer is still one of the most common cancers among females^1^. In China, there was an estimated 989,000 new cases and 305,000 deaths in 2015^2^. Treatment options for cervical cancer are usually based on The International Federation of Gynecology and Obstetrics (FIGO) staging system. However, unlike other gynecological cancers^3^, the former FIGO staging system of cervical cancer doesn’t include regional lymph node status^4^. Which means that lymph node metastasis won’t change the stage of patients.

Lymph node metastasis has been identified as an important prognostic factor affecting survival outcomes for cervical cancer in many studies^5–8^. Singh Ak, *et al*.^8^ showed that cervical cancer patients with positive para-aortic lymph nodes and pelvic lymph nodes had significant inferior 3-year cause specific survival (CSS) than those without lymph node metastasis (29% vs 73%, P = 0.0005). In our previous study^7^, Wang W, *et al*. reviewed 1433 patients with cervical cancer treated with definitive radiotherapy, the 3-year disease free survival (DFS) for patients with and without regional lymph node metastasis were 58.0% and 81.8% (P < 0.001), respectively.

Considering the adverse affection of regional lymph node metastasis and other factors^9^ on survival of cervical cancer. In 2018, The International Federation of Gynecology and Obstetrics (FIGO) revised the 2014 staging system of cervical cancer^10^. One important change from the previous staging system is that the new staging system designates patients with regional lymph node metastasis into stage III. Patients with pelvic lymph node metastasis only are defined as stage IIIC1. Stage IIIC2 includes patients with positive para-aortic lymph node.

One thing to be aware of is that stage IIIC1 of the new staging system doesn’t take primary tumor size and extent into consideration. While tumor size was associated with survival outcomes in previous study^9^. This strongly indicates that patients with stage IIIC1 are not homogeneous. Further risk stratification is needed for this group of patients.

The objective of this study was to investigate the heterogeneity of patients with stage IIIC1 and conduct a risk stratification based on prognostic factors for DFS in patients with stage IIIC1.

## Materials and Methods

### Patient selected

After obtaining institutional review board approval from Peking Union Medical College Hospital. We reviewed the clinical data of patients with cervical cancer treated with definitive intensity-modulated radiotherapy (IMRT) at our institute between January 2008 and December 2014. The inclusion criteria were as follows: biopsy proven cervical cancer; FIGO stage III1 based on the 2018 revised FIGO staging system; with exhaustive imaging records including thoracic and abdominal computed tomography (CT), Pelvic magnetic resonance imaging (MRI) or positron emission tomography/computed tomography (PET/CT); no evidence of distant metastasis before treatment; no previous treatment of cervical cancer. As described in our previous articles^11^, lymph node metastasis was diagnosed by imaging. For patients who received PET/CT, pelvic and para-aortic lymph nodes with FDG accumulation greater than liver accumulation or standard uptake value (SUV) > 2.5 ng/ml were identified as positive lymph nodes. For patients without PET/CT, lymph nodes with the short axis diameter longer than 1 cm were defined as lymph nodes metastasis.

### Radiotherapy

The detailed information of radiotherapy was described in our previous studies^6,7,12^. In brief, all patients in our study received IMRT to whole pelvic cavity and intracavity brachytherapy. Clinical target volume (CTV) included the primary tumor, uterus, cervix, parametrium, part of the vagina (depending on the extent of the primary tumor) and pelvic lymphatic drainage area (including common iliac, internal iliac, external iliac, obturator and presacral lymph node regions). Margins of 7–10 mm was added to CTV to form the planning clinical target volume (PCTV). A dose of 50.4 Gy in 28 fractions was prescribed to at least 95% of PCTV. Positive pelvic lymph nodes were defined as gross tumor volume (GTVnd). GTVnd plus a 5 mm margin was defined as planning gross tumor volume (PGTVnd). At least 95% of the PGTVnd received a dose of 59–61 Gy in 28 fractions with simultaneous integrated boost (SIB).

All patients received intracavity brachytherapy with ^192^Ir, a total dose of 30–36 Gy in 5 to 7 fractions was delivered to point A.

### Chemotherapy

Cisplatin (30–40 mg/m^2^ per week) was the first line regimen for concurrent chemotherapy. For patients with renal failure, paclitaxel (60–80 mg/m^2^ per week) was an alternative choice. No patients received neoadjuvant or adjuvant chemotherapy in our institute.

### Follow-up

Patients received first follow-up examination one month after treatment. Then, every three months in the first two years, every six months during the third to the fifth year, once a year thereafter. Routine examinations included gynecological examinations, squamous cell carcinoma antigen (SccAg), thoracic and CT, pelvic MRI. Patients would receive PET/CT examination if they were suspected of disease relapse.

### Methodology and statistical analyses

Among patients met the inclusion criteria, characteristics of patients including age, histology, tumor size, tumor extension, status of pelvic lymph node metastasis (PLNM), number of PLNM and treatment type were extracted from the database.

We chose DFS as the end point of our study. DFS was defined as time from date of the start of treatment to date of disease progression (local recurrence or distant metastasis) or last follow up. DFS was calculated with Kaplan-Meier method.

Tumor size, parametrial infiltration, invasion of pelvic wall, invasion of the lower third of vagina, bilateral PLNM, common iliac lymph node metastasis and number of PLNM were chosen as potential significant factors affecting DFS. The optimal cut-off values of tumor size and number of PLNM were obtained by receiver operating characteristic (ROC) curves^13^. The ROC curves depicted time-dependent area under the curve with tumor size, number of PLNM and 3-year DFS, respectively. The cut-off values were derived with the Youden index that maximizes the sum of sensitivity and specificity. Univariate analysis with log-rank test and Multivariate analysis with cox proportional hazard model were used to confirm the significant adverse factors for DFS in patients with stage IIIC1.

A risk stratification based on identified prognostic factors was conducted for patients with cervical cancer in stage IIIC1. DFS of different risk groups were compared with log-rank test.

All statistical analyses were performed with SPSS 23.0. Statistical difference was defined as a two-side P value of <0.05.

### Ethical approval

All procedures performed in studies involving human participants were in accordance with the ethical standards of the institutional and/or national research committee and with the 1964 Declaration of Helsinki and its later amendments or comparable ethical standards.

### Informed consent

Informed consent was obtained from all individual participants included in the study.

## Results

### Patient and tumor characteristics

Between January 2008 and December 2014, a total 1433 patients with biopsy proven cervical cancer were treated with IMRT at our institute. After re-staging with the 2018 FIGO staging system, three hundred and twenty-five patients were categorised into FIGO stage IIIC1. These 325 patients constituted our study group.

Table 1 shows the characteristics of patients with FIGO stage IIIC1 (2018). The optimal cut-off value for tumor size and number of PLNM were 4 cm and two based on the aforementioned ROC curves. Patients with FIGO stage IIIC1 were prone to have large tumors (240/325, 73.9%) and parametrial infiltration (265/325, 81.5%). Patients with bilateral PLNM accounted for 44.6% (145/325) of all patients in stage IIIC1. Common iliac lymph node metastasis was discovered in 49 patients (15.1%). One hundred and seventy-one patients (52.6%) had at least two positive pelvic lymph nodes.

**Table 1:** Characteristics of patients with FIGO stage IIIC1 (2018).

Characteristics	No. (100%)
Total	325
Age (years old)	
Median	50
<30	5 (1.6%)
30–39	26 (8.0%)
40–49	131 (40.3%)
50–59	118 (36.3%)
60–69	39 (12%)
≥70	6 (1.8%)
Histology	
Scc	301 (92.6%)
non-Scc	21 (6.5%)
unclear	3 (0.9%)
Tumor size	
<4 cm	80 (24.6%)
≥4 cm	240 (73.9%)
unclear	5 (1.5%)
Parametrial infiltration	
yes	265 (81.5%)
No	60 (18.5%)
Invasion of pelvic wall	
yes	60 (18.5%)
No	265 (81.5%)
Invasion of the lower third of vagina	
Yes	11 (3.4%)
No	314 (96.6%)
Pelvic lymph node metastasis	
bilateral	145 (44.6%)
unilateral	135 (41.5%)
uncertainty	45 (13.9%)
Common iliac lymph node metastasis	
yes	49 (15.1%)
no	231 (71.1%)
uncertainty	45 (13.8%)
Number of PLNM	
≥2	171 (52.6%)
<2	109 (33.5%)
uncertainty	45 (13.9%)
CCT	
yes	288 (88.6%)
no	37 (11.4%)

Abbreviations: FIGO = International Federation of Gynecology and Obstetrics, Scc = squamous cell carcinoma, CCT = concurrent chemotherapy, PLNM = pelvic lymph node metastasis.

**Figure 1:**
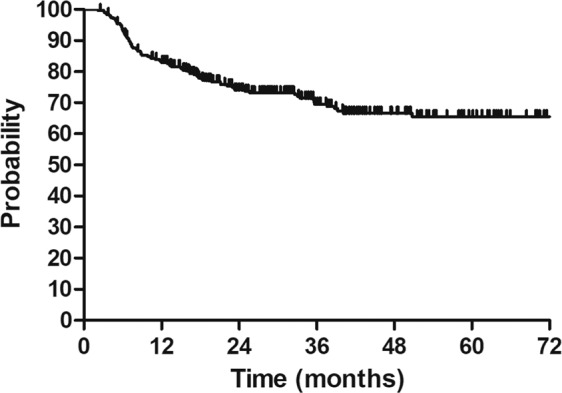
Disease free survival (DFS) for patients with cervical cancer in stage IIIC1 (FIGO 2018). 3-y DFS: 66.3%.

The median follow-up duration was 28.4 months (range: 1.9–114.2 months). The 3-year DFS was 66.3% for the 325 eligible patients (Fig. 1).

### Univariate and multivariate analyses

**Figure 2:**
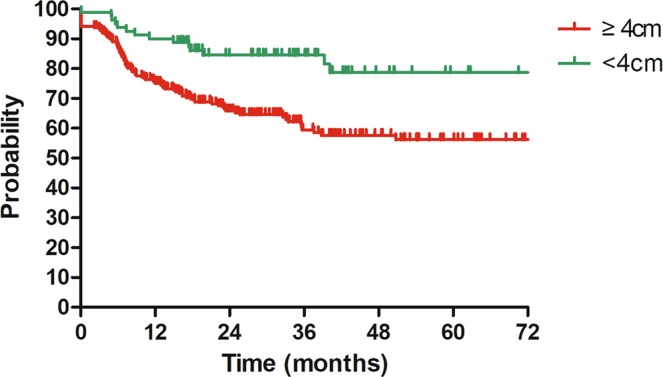
Disease free survival (DFS) for patients with cervical cancer in stage IIIC1 (FIGO 2018) regarding rumor size. 3y-DFS: ≥4 cm vs <4 cm = 57.6% vs 84.5% (HR = 2.00, 95%CI = 1.13–3.57, P = 0.018).

**Figure 3:**
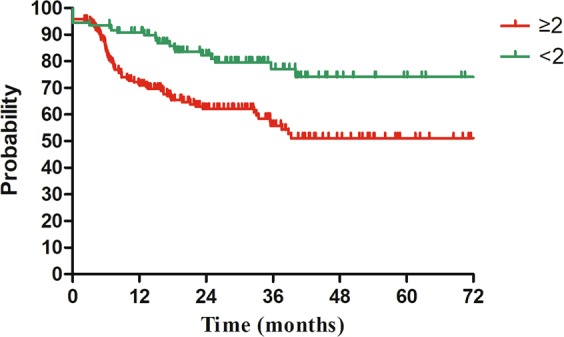
Disease free survival (DFS) for patients with stage IIIC1 (FIGO 2018) regarding number of pelvic lymph node metastasis. 3y-DFS: ≥2 vs <2 = 55.8% vs 79.6% (HR = 2.10, 95%CI = 1.04–4.24, P = 0.039).

On univariate analysis, the 3-year DFS varied significantly regarding tumor size, invasion of pelvic wall, bilateral lymph node metastasis and number of PLNM for patients with stage IIIC1 (2018). While on multivariate analysis, tumor size and number of PLNM remained significantly associated with DFS. The 3-year DFS for patients with tumor size of ≥4 cm and <4 cm were 57.6% and 84.5%, respectively (HR = 2.00, 95%CI: 1.13–3.57, P = 0.018, Fig. 2). Patients with at least two positive pelvic lymph nodes experienced worse 3-year DFS than those with only one positive pelvic lymph node (55.8% vs 79.6%, HR = 2.10, 95%CI: 1.04–4.24, P = 0.039, Fig. 3). The detailed information of univariate and multivariate analyses is shown in Table 2.

**Table 2:** Univariate and multivariate analysis for DFS in patients with FIGO IIIC1 (2018).

Variables	No. of patients 325	3-year DFS	Univariate analysis (Log-rank test)	Multivariate analysis (Cox proportional hazard model)	
			P	HR (95%CI)	P
Tumor size					
≥4 cm	240 (73.9%)	57.6%	**0.001**	2.00 (1.13–3.57)	**0.018**
<4 cm	80 (24.6%)	84.5%			
uncertainty	5 (1.5%)				
Parametrial infiltration					
yes	265 (81.5%)	65.1%	0.589		
no	60 (18.5%)	69.4%			
Invasion of pelvic wall					
yes	60 (18.5%)	51.1%	<**0.001**	0.65 (0.40–1.05)	0.078
no	265 (81.5%)	69.9%			
Invasion of the lower third of vagina					
Yes	11 (3.4%)	45.5%	0.084		
no	314 (96.6%)	69.1%			
Pelvic lymph node metastasis					
bilateral	145 (44.6%)	54.6%	**0.005**	1.06 (0.57–1.97)	0.855
unilateral	135 (41.5%)	72.4%			
uncertainty	45 (13.9%)				
Common iliac lymph node metastasis					
yes	49 (15.1%)	57.8%	0.187		
no	231 (71.1%)	65.3%			
uncertainty	45 (13.8%)				
Number of PLNM					
≥2	171 (52.6%)	55.8%	<**0.001**	2.10 (1.04–4.24)	**0.039**
<2	109 (33.5%)	79.6%			
uncertainty	45 (13.9%)				

Abbreviations: FIGO = International Federation of Gynecology and Obstetrics, HR = hazard ratio, DFS = disease free survival, PLNM = Pelvic lymph nodes metastasis.

### Risk stratification

**Figure 4:**
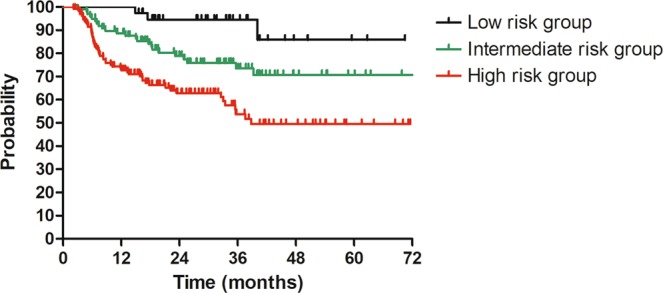
Disease free survival (DFS) for patients with stage IIIC1 (FIGO 2018) regarding risk groups. 3y-DFS: low-risk vs intermediate-risk vs high-risk = 51.1% vs 70.0% vs 92.1% (P < 0.001).

Based on the identified prognostic factors for DFS, we conducted a risk stratification for patients with stage IIIC1. Patients with zero, one, and two prognostic factors were designated into low-risk, intermediate-risk and high-risk group, respectively. After stratification, Low-risk group included 39 patients, one hundred and three patients contributed to intermediate-risk group and 138 patients constituted high-risk group. The remaining 45 patients didn’t receive stratification due to lacking in data of lymph node metastasis number. There was a significant difference regarding DFS among different risk groups. The 3-year DFS for patients in low-risk, intermediate-risk, and high-risk group were 92.1%, 70.0%, and 51.1%, respectively (P < 0.001, Fig. 4).

## Discussion

After the 2018 FIGO staging system for cervical cancer was released, Matusuo, K and colleagues performed a validation analysis with data from The Surveillance, Epidemiology, and End Results (SEER)^14^. They found that the 5-year cause-specific survival for patients with stage IIIC1 (2018) varied widely from 39.3% to 74.8% by tumor size. This finding and previous study^9^ suggested us pay attention to the heterogeneity of this group of patients.

In our study, univariate and multivariate analyses regarding DFS for patients with stage IIIC1 were performed. After multivariate analysis, tumor size was illustrated to significantly influence DFS. Patients with large tumors (≥4 cm) experienced worse 3-year DFS than those with tumor size of <4 cm (57.6% vs 84.5%, HR = 2.00, 95%CI: 1.13–3.57, P = 0.018, Fig. 2). These 3-year DFS were comparable to stage IIIB (59.8%) and IIA (84.0%) reported previously^7^. Our finding further highlights the effect of local tumor factors on the survival of cervical cancer patients with stage IIIC1.

The influence of characteristics of lymph node metastasis on survival for patients with cervical cancer has been investigated in several studies^15–17^. Okazawa M, *et al*.^15^ found that patients with multiple pelvic lymph nodes metastasis (≥3) experienced significantly worse progression free survival (PFS) than those with 1 or 2 pelvic lymph nodes metastasis. The size of lymph node significantly affected survival of patients with cervical cancer in the study of Song S, *et al*.^16^. The 5-year DFS for patients with negative lymph node, small lymph node (<15 mm) and large lymph node (≥15 mm) were 80%, 67% and 50%, respectively (P < 0.001). Except for LN-number and LN-size. Li X, *et al*.^17^ also showed a significant relationship between LN-volume, matted/necrotic LN and survival outcomes in patients with cervical cancer. In our study, characteristics of lymph node metastasis included bilateral PLNM, common iliac lymph node metastasis and number of PLNM. Number of PLNM finally showed to be related with DFS. Patients with two or more positive pelvic lymph nodes had about two-hold increased risk of disease progression, when compared to patients with only one positive pelvic lymph node (HR = 2.10, 95%CI: 1.04–4.24, P = 0.039). This finding, together with aforementioned studies confirms that the characteristics of lymph node metastasis plays an important role on the survival outcomes of cervical cancer patients with stage IIIC1.

Our study identified two significant prognostic factors including tumor size and number of PLNM for DFS in cervical cancer patients with stage IIIC1. The 2018 FIGO staging system has already included tumor size in stage IA, IB and IIA. For patients with stage IIIC1, a further grouping based on tumor size may be reasonable. In other pelvic cancers, such as rectal cancer, number of lymph node metastasis is a relevant factor in the staging system. Patients with one to three positive regional lymph nodes are staged into N1, stage N2 includes patients with four or more regional lymph node metastasis. Since number of PLNM significantly affects the survival outcomes of cervical cancer patients with stage IIIC1, it would be considerable to include number of PLNM in the next revision of FIGO staging system for cervical cancer.

Based on the prognostic factors for DFS, we performed a risk stratification for patients with stage IIIC1. Patients were divided into low, intermediate, and high-risk groups by number of prognostic factors. A significant difference for DFS was found among different subgroups. The 3-year DFS was 92.1%, 70.0% and 51.1% for patients in low, intermediate and high-risk groups, respectively (P < 0.001). Considering the poor survival for patients in high-risk group, more intense treatment, such as adjuvant chemotherapy may be needed for this group of patients^18^. Surprisingly, we found that the 3-year DFS for patients in low-risk group was as high as 92.1%, which was quite comparable to that of FIGO IB patients we reported previously^7^. For this group of patients, maybe a redefinition of stage for them is needed.

As far as we know, our study is the first one to investigate the heterogeneity of patients with stage IIIC1 of the 2018 FIGO staging system for cervical cancer, and we successfully stratify patients into three subgroups. However, there are still several limitations. First, this is a retrospective study from a single institution and several clinical data were missing because of its retrospective nature. Moreover, due to lacking in clinical records of patients with cervical cancer who received surgery, patients with stage IIIC1 who received surgery were not involved in our study.

In conclusion, our study confirms the heterogeneity of patients with cervical cancer in FIGO stage IIIC1 (the 2018 FIGO staging system). Tumor size and number of PLNM are significant prognostic factors for DFS in patients with stage IIIC1. The next revision of FIGO staging system for cervical cancer, especially for stage IIIC1, should focus on tumor size and number of pelvic lymph node metastasis.

## Data Availability

The data is available by request at the corresponding authors.
